# Integrating evidence into policy and sustainable disability services delivery in western New South Wales, Australia: the 'wobbly hub and double spokes' project

**DOI:** 10.1186/1472-6963-12-70

**Published:** 2012-03-21

**Authors:** Craig Veitch, Michelle Lincoln, Anita Bundy, Gisselle Gallego, Angela Dew, Kim Bulkeley, Jennie Brentnall, Scott Griffiths

**Affiliations:** 1 Faculty of Health Sciences, University of Sydney, Cumberland Campus C42, PO Box 170, Lidcombe 1825, NSW, Australia; 2 School of Medicine, Building 30, Campbelltown Campus, University of Western Sydney, Locked Bag 1797, Penrith 2751, NSW, Australia; 3 NSW Government - Ageing, Disability & Home Care, PO Box 865, Dubbo 2830, NSW, Australia

**Keywords:** Rural, Remote, Regional, Allied health, Disability, Workforce, Retention, Policy, Service provision, Access

## Abstract

**Background:**

Policy that supports rural allied health service delivery is important given the shortage of services outside of Australian metropolitan centres. The shortage of allied health professionals means that rural clinicians work long hours and have little peer or service support. Service delivery to rural and remote communities is further complicated because relatively small numbers of clients are dispersed over large geographic areas. The aim of this five-year multi-stage project is to generate evidence to confirm and develop evidence-based policies and to evaluate their implementation in procedures that allow a regional allied health workforce to more expeditiously respond to disability service need in regional New South Wales, Australia.

**Methods/Design:**

The project consists of four inter-related stages that together constitute a full policy cycle. It uses mixed quantitative and qualitative methods, guided by key policy concerns such as: access, complexity, cost, distribution of benefits, timeliness, effectiveness, equity, policy consistency, and community and political acceptability.

Stage 1 adopts a policy analysis approach in which existing relevant policies and related documentation will be collected and reviewed. Policy-makers and senior managers within the region and in central offices will be interviewed about issues that influence policy development and implementation.

Stage 2 uses a mixed methods approach to collecting information from allied health professionals, clients, and carers. Focus groups and interviews will explore issues related to providing and receiving allied health services. Discrete Choice Experiments will elicit staff and client/carer preferences.

Stage 3 synthesises Stage 1 and 2 findings with reference to the key policy issues to develop and implement policies and procedures to establish several innovative regional workforce and service provision projects.

Stage 4 uses mixed methods to monitor and evaluate the implementation and impact of new or adapted policies that arise from the preceding stages.

**Discussion:**

The project will provide policy makers with research evidence to support consideration of the complex balance between: (i) the equitable allocation of scarce resources; (ii) the intent of current eligibility and prioritisation policies; (iii) workforce constraints (and strengths); and (iv) the most effective, evidence-based clinical practice.

## Background

As with other health professions worldwide, the Australian allied health workforce is in short supply and not well distributed [1,2]. Policy that supports rural health service delivery is important given the shortage of allied health services outside of Australian metropolitan centres [1]. The shortage of allied health professionals means that those in rural areas work long hours and have little peer or service support [3]. Service delivery to rural and remote communities is further complicated because relatively small numbers of clients are dispersed over large geographic areas [3].

### Need for workforce policy to support rural allied health service delivery

Allied health professionals such as physiotherapists, occupational therapists, speech pathologists and psychologists assist people to participate fully in their families, employment and communities. Delays in access to, and poor coordination of, services mean that problems often compound and that secondary complications arise, resulting in increased need for services [4]. Unmet needs also result in reduced participation in family and community life, with flow-on social and economic costs for individuals, families and communities from missed opportunities and lost income. In short, the dearth of rural allied health services represents a substantial problem.

Three service delivery models characterise most allied health services in rural communities: (i) service teams located in regional centres that meet local needs but require more distant clients to travel; (ii) service teams from regional centres that travel to smaller communities to provide outreach services; and (iii) urban-based 'fly-in and fly-out' services provided to selected rural communities. A fourth model, 'hub and spoke', is an extension of the outreach services model where outreach staff in several remote locations ('spokes') are supported by a regional centre 'hub'. The spokes bring staff closer to clients, reducing travel time and costs and increasing staff knowledge of and connection to the community. Meanwhile, the hub is a large enough centre to support staff from multiple spokes administratively and with professional development opportunities and collegial relationships [3,5,6].

In Australia, the allied health workforce falls into two broad sectors: health and disability. Allied health professionals in the health sector are generally located in hospitals, primary health care services, acute rehabilitation services, and private practice. In the disability sector allied health professionals (therapists) work within government services, non-government agencies, schools, community, and private practice. In New South Wales (NSW), disability services are planned, funded and supported by the NSW Government Family & Community Services - Ageing, Disability and Home Care (ADHC), which is ministerially and administratively separate from the state health department (NSWHealth). Disability services provide allied health therapy, case management, behaviour support, accommodation, employment, day services, respite care, information and advocacy across the life span to people with a disability [7]. This project focuses primarily on allied health professionals (therapists) who provide services to people with disabilities in rural and remote areas in western NSW.

### Maximising the potential of the rural allied health workforce

The problem of continuing allied health workforce insufficiency is one to which ADHC-Western Region (ADHC-WR) has applied various strategies. Staff retention is a core element of sustainable health service delivery [3]. One pragmatic strategy used by ADHC-WR is to appoint appropriately skilled therapists, regardless of their location within the region. Under this model (locally dubbed the 'wobbly hub'), team members come together around clients and administrative requirements rather than working together as formal teams in particular locations or 'on the road'. This enables therapy services to be provided in areas where there are no resident therapists.

ADHC-WR aims to provide interdisciplinary therapy services to clients wherever possible, as best practice, to maximise effectiveness and to minimise overlaps/gaps [8], particularly as most ADHC-WR clients have multiple service needs [9]. Interdisciplinary teams are most effective when they are able to see clients at the same time [5]. However, the current wobbly hub distribution of the allied health workforce is not conducive to this teamwork occurring regularly. ADHC-WR senior clinicians also find that implementation of their current *Intake Policy (2001) *and *Prioritisation and Allocation Policy (2001) *[internal documents not publicly available], while effective in addressing issues of resource limitations, does not facilitate a team approach. A proposed extension to the wobbly hub arrangement, which involves clients being transported to therapy services (dubbed 'double spoke' by ADHC-WR staff), appears to provide a partial solution to redress this situation by decreasing travel time for therapists, increasing frequency of services, and enabling interdisciplinary practice. This kind of arrangement has not previously been described. It will be explored as an innovative aspect of this project to better understand its benefits and limitations and how these can be encompassed in policy.

### A partnership approach to developing evidence-based workforce policy

Policy makers continually confront a tension between competing demands (eg financial and political imperatives), and absent or conflicting research evidence for effectiveness [10]. Clinicians often feel caught between policy and their vision of their primary role in meeting the needs of clients and families, with implications for job satisfaction. Research required for evidence-based policy must therefore be multi-faceted to support the generation of policy and promote its effective implementation. Additionally, policy makers require policy-useable evidence to be quickly and readily available and often criticise the substantial time-lag between the conduct of academic research and availability of policy-useable evidence.

This project, funded under the National Health & Medical Research Council Partnerships for Better Health scheme, includes academic researchers, policy makers and clinicians. The core tenets of the partnership are: clinicians, clients and carers are the primary knowledge-holders about service delivery and use; academic researchers possess expertise and techniques to collect, analyse, interpret and recommend appropriate information rigorously; and policy makers possess understanding of policy imperatives and policy development experience [11]. The expectation is that by working together in a cycle of generating and interpreting evidence, developing and applying evidence-based policy, and evaluating outcomes will result in timely, evidence-based policy and the best service delivery decisions.

### Aims

The project has two main aims, with a number of objectives to achieve these:

1. Generate a sustainable process of timely evidence collection and policy evaluation and development in ADHC-WR

o Identify service evaluation measures that are both clinically- and policy-relevant

o Develop policy makers', managers', and senior clinicians' skills to conduct, interpret and use research evidence relevant to policy development

o Build an evidence-based culture in policy development for allied health service delivery

2. Complete a cycle of policy evaluation and development for allied health service delivery in ADHC-WR

o Provide evidence regarding the impact of ADHC-WR's current mix and models of allied health service delivery using a range of multilevel measures (economic, service, staff and client)

o Identify policy-relevant issues of importance to clinicians in health service delivery, and to client/carers with respect to health service utilisation

o Assess the impact of a collaboratively developed model of allied health service delivery using a range of multilevel measures

o Evaluate the impact of new or modified allied health service delivery policies using a range of multilevel measures

## Methods/Design

### Setting

The region under study accounts for 72% of the 800,642 square kilometre land area of New South Wales, stretching from the state's borders with Victoria in the south, South Australia in the west, Queensland in the north, and to the Great Dividing Range in the east. In 2009, the estimated resident population was nearly 604,000 [12]. The region's population is dispersed among large regional towns with populations of 20-40,000, smaller towns of 1-3,000, and isolated rural communities of 1,000 or fewer. Some people live on remote properties (farms) many kilometres from their nearest neighbours and hundreds of kilometres from towns. At the 2006 Census, the region's population accounted for 9% of the state's population. People aged over 65 years accounted for 14.9% of the region's population, compared with 13.8% for the state as a whole. Indigenous people accounted for 5.6% of the region's population compared with 2.1% for the state as a whole. People with a disability accounted for 7.2% of the region's population. Two in every three of the region's people with a disability required assistance, of some sort, with activities. [Data supplied by NSW Government Family and Community Services - Ageing, Disability and Home Care, derived from Australian Bureau of Statistics 2006 Census data.]

### Overall study design

**Figure 1:**
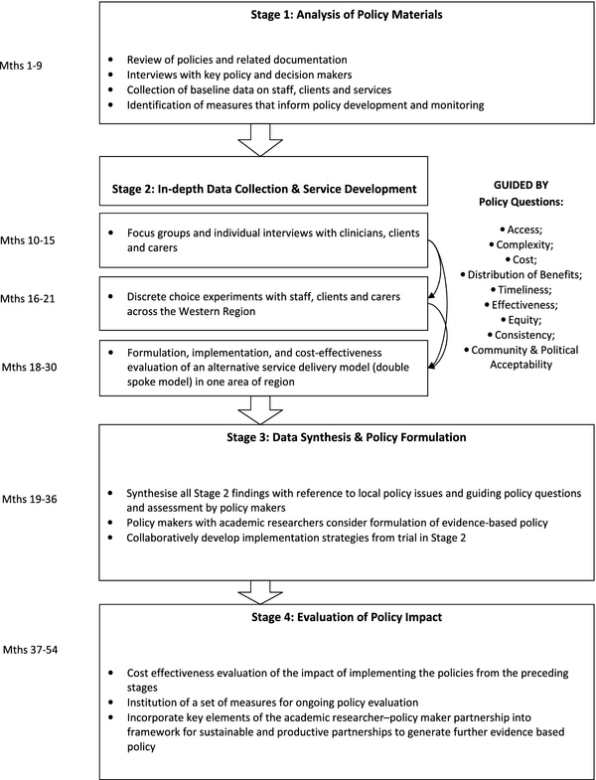
**Outline of Project Stages**.

There are four inter-related stages that together constitute a full policy cycle [13] (see Figure 1). We will use mixed quantitative and qualitative methods, guided by key policy concerns: access, complexity, cost, distribution of benefits, timeliness, effectiveness, equity, policy consistency, and community and political acceptability [14]. Ethics approval has been granted by the University of Sydney Human Research Ethics Committee (#*10-2009/12194)*. Written consent will be obtained from all study participants. All interviews will be anonymised. All data will be kept confidential.

#### Stage 1: Analysis of policy materials

Stage 1 adopts a policy analysis approach in which existing relevant policies and related documentation will be collected and reviewed. The purpose of the review is to gain insight into the issues currently influencing policy development and implementation. Additionally, gaps in key policy-related evidence will be identified. Relevant documents and operational guidelines providing current direction to staff working in government disability services in NSW spanning the years 1993 (introduction of the Disability Act [7]) to 2016 (extent of current *Stronger Together *initiative [15]) will be identified and collected using purposeful [16] and snowballing [17] sampling techniques.

Each document will be read and a summary of the key policy content made. This initial summary will be used to assign documents into three tiers. Tier 1: overarching NSW Government policy documents including major disability-specific and general population related documents. Tier 2: ADHC specific policies, sub-divided according to whether they are client-focussed or workforce-focussed. Tier 3: ADHC operational guidelines also sub-divided into client-focussed and workforce-focussed. The collation and qualitative content analysis [17] of these documents will generate themes that will guide individual interviews.

Key policy makers and management staff within ADHC (both central office and Western Region) will be interviewed regarding issues that influence policy development and implementation. Interviews will be audio-recorded with participants' permission. The information will be transcribed and analysed to identify key issues and variables for exploration in Stage 2. Purposeful sampling [16] will be used to identify approximately 50 senior staff employed either by ADHC or by non-ADHC agencies supporting service delivery to people with a disability in western NSW. Senior staff include executive team members, planning team members, senior therapists who provide clinical supervision to therapists, and senior managers overseeing community support staff including therapists. ADHC central office staff include senior therapy practice leaders, and the senior managers overseeing relevant directorates. Non-ADHC disability and health agencies include managers of agencies based in western NSW, and managers of services delivered in western NSW via outreach from head offices in Sydney (capital city of NSW).

Comprehensive summary notes derived from the audio-recordings of the interviews will be sent to participants' for review and comment [17]. Summaries will be analysed using thematic analysis to identify the main issues around policy development and implementation [18].

Concurrently, baseline data regarding staff, clients and services will be collected for current service delivery models. We will identify measures that may inform policy development and monitoring in line with the issues raised. These will include those already collected, as well as new measures that might feasibly be implemented (examples are given under 'Data' below.) These data will be collected at 6-monthly intervals throughout the project duration.

#### Stage 2: In-depth data collection and service development

Stage 2 uses a mixed methods approach to collecting information from allied health professionals and service users (clients and their carers). The focus will be three-fold: (i) issues identified in Stage 1; (ii) other issues of particular importance in terms of providing and receiving allied health services that might influence policy development; and (iii) variables that will inform the development and interpretation of Discrete Choice Experiments (DCEs) to elicit staff and servicer user preferences. These in-depth data will assist understanding of the current policy implementation and issues arising; and capture the expertise of clinicians and managers to identify barriers, formulate alternatives, and implement change.

Stage 2a will begin with a series of focus groups and individual interviews with ADHC staff, allied health professionals, service users, non-ADHC agencies and other community organisations to gather baseline information on service delivery and gaps, and perceptions and expectations of relevant policy (approximately 50 service providers and approximately 100 service users). Focus groups will be used to identify key issues by consensus. Focus groups provide an opportunity for people with similar experiences to share information in a time- and cost-effective way [16,19]. A group dynamic also occurs as a result of the interaction between focus group participants as they both explain their own position to the group and question others' points of view [20]. Attendance at a focus group will not be feasible for all participants given their geographic spread across western NSW, or their availability at the time the researchers visit a particular geographic location. Therefore, individual interviews will be offered to participants unable to attend a focus group. Interviews will cover the same topics as focus groups. Two investigators (AD and KB) will conduct all focus groups and interviews.

Focus group and individual interviews data will be transcribed and analysed as in Stage 1. Information from focus groups and individual interviews will be synthesised during analysis using a modified grounded theory approach to identify themes. Constant comparison will be used within and across data sets to identify commonalities and differences [21,22].

Stage 2b involves the development and conduct of surveys incorporating discrete choice experiments (DCEs) to elicit allied health staff and service user preferences across the Western Region [23,24]. The DCEs will be developed from data collected in the policy analysis and qualitative phase. The staff DCE will enable the estimation of the relative importance that staff members place on different work (job) characteristics and trade-offs they are willing to make between components. The service user DCE will identify the trade-offs that service users are willing to make in terms of service design and access. The DCEs will then be used to forecast choices or option values that can inform ADHC-WR workforce and service delivery policies.

DCE methodology is a process which determines the comparative or relative value that people place on a set of factors (attributes) [25,26]. Its application in health care settings is growing rapidly and is now an accepted method of valuing the benefits of health care services [24]. The method's particular strength is that it enables determination of what people are prepared to forego in order to maintain or obtain something else [25]. It is firmly based in economic choice theory yet uses an intuitive and easily understandable approach to examine the trade-offs between attributes of a health service. There is a growing number of studies that use the DCE method to assess service preferences [23,24,27] or treatment preferences [28]. The basic premise on which a DCE is based is that a service can be described in terms of a number of attributes that can be set at different levels [29]. Participants are asked to choose between different service options, each of which is composed of different permutations of the attributes. The underlying assumption is that (economically) rational individuals will always choose the good or service with the highest value or level of utility to them [30].

The DCE will be constructed using a fractional factorial experimental design. With a fixed number of attributes and levels for each attribute, there are a very large number of potential permutations. For example, with six attributes and three levels each there are 3^6 ^= 729 possible combinations. This is clearly too many to present to one individual, so the number of potential scenarios is reduced to a more manageable number, usually 16, using processes such as the D-optimal design method. This ensures that the levels are varied so they are orthogonal and satisfy statistical properties such that the results do not depend on how the attribute levels were combined [26]. Each DCE questionnaire will be piloted on 20 out-of-region participants - ie 20 therapists and 20 service users from other rural regions of NSW.

Sample size in DCEs is usually based on having sufficient individuals (between 30 and 100) within each sub-group. The therapist DCE will be limited to 3 sub-groups (eg discipline, location, time in region), implying a minimum sample of 90 respondents. There are approximately 100 therapists in the region, thus all therapists in the region will be invited to participate in the survey. The service user DCE will involve up to six sub-groups in total, implying a minimum sample of 180 respondents. It will be distributed to a locationally-stratified random selection of approximately 200 service users. As the population is widely distributed across the region, locational stratification will be employed to ensure a sample is selected that represents the range of locations, and travel distances. Stratification will likely encompass common travel distance/time thresholds for seeking disability services within the region (eg within town, within 50 kilometres of service, 50-100 kilometres to service, > 100 kilometres of service [31]).

Analyses will include summary and descriptive statistics, as well as comparative analyses using relevant parametric and non-parametric tests. In brief, the analysis of discrete choice data is based on random utility models [32], which are derived under the assumption of utility maximizing behaviour by the decision-maker. A random parameter or a Multinomial Mixed Logit model (MMNL- also known as random parameters logit RPL) will be estimated to explore the job (for therapists) and service (for service users) preferences and to reveal the order of these or relative influence of each attribute. Additionally, associations between covariates such as socio-demographics and job/service attributes will be investigated in the mixed logit analysis.

Stage 2c will draw on ADHC-WR staff experiences and local requirements (including new data from Stage 1 and Stage 2a/b), to formulate and implement an alternative service delivery model in one area of the region. We will implement and further assess the policy-informing utility of the measures identified in Stage 1. We also will collect focused economic and outcome information to evaluate the cost-effectiveness of the model by comparing data with baseline data from Stage 1. The most relevant measures will be instituted into practice and used in service monitoring and policy development in Stage 4 (and beyond, following the completion of the project).

#### Stage 3: Data synthesis and policy formulation

This stage focuses on two issues: (i) identifying and implementing evidence that will best inform policy; and (ii) continuing the process of mapping the academic researcher - policy maker partnership to enable researchers to provide timely policy-relevant information to policy makers.

All findings from Stages 1 and 2 will be synthesised with reference to the key policy issues raised by the partners and guiding policy questions [14] (see Figure 1). These will be assessed by an independent group of policy makers from ADHC and other organisations in terms of validity, utility, and timeliness in policy development. From this, the partnership team will distil the most relevant evidence (including outcome measures) to aid policy development and implementation.

The team will then work collaboratively to make the transition from evidence to evidence-based policy. In this Stage the policy maker partners will take the lead, with the academic researchers assisting to interpret, extend and add detail to the evidence. The team will also use the results of the implementation of the alternative service delivery model (from Stage 2c) to develop strategies for the implementation of evidence-based policy across the Western Region.

#### Stage 4: Evaluation of policy impact

This stage will monitor and evaluate the implementation and impact of new or adapted policies that arise from the preceding stages across a 12-18 month period. The process will include an assessment of the reliability and utility of specific outcome measures instituted into practice during Stage 2. The collection of specific economic data will continue in order to assess the cost implications of those policies. This Stage will focus on sustainability and handing the process on to the policy maker partners.

Focus group sessions will again be conducted with (i) allied health professionals to elicit their experiences of the policies and the monitoring measures; (ii) ADHC-WR and non-ADHC managerial staff to elicit their experiences of the policies and the monitoring measures; and (iii) service users to elicit their experiences and opinions of changes to service delivery. Similar numbers of participants will be recruited and data analysed in the same ways as outlined for Stages 1 and 2.

### Secondary data and policy analysis

o Demographics, population/client distribution, disability and service availability data (eg Australian Bureau of Statistics and Australian Institute of Health and Welfare data, National Disability Administrators' Small area estimates [33], service registers and waiting lists)

o Policies and related documentation (eg policies and local adaptations to policy, published policy guidelines, documented procedures and actions to implement policy)

### Service data

o Quantitative (eg frequency, length, and number of sessions per episode of care)

o Descriptive data on type or combination of services and goals addressed (eg coding on the International Classification of Functioning, Disability and Health (ICF) [34])

o Costs of service provision (eg staff time, travel and accommodation, client travel)

o Group outcomes for clients and families on global measures (eg Goal Attainment Scaling [35], ICF)

o Achievement of client-/family-centred service mission and policy (eg quantitative Measure of Process of Care [36])

### Focus groups and individual interviews

o Managers' and decision-makers' perceptions of the drivers of and needs for acceptable, timely policy, and current and priority issues and constraints

o Allied health professionals' qualitative perceptions of opportunities and opportunity costs, drivers of team processes (eg need, timetabling, geography, position vacancies), and satisfaction with work

o Service users' experiences of policy in action: qualitative perceptions of opportunities and opportunity costs, and the congruence between short-term service goals and meaningful long-term outcomes

### Discrete choice experiments (DCEs)

o Clinicians: to identify the relative importance that clinicians place on different work characteristics and trade-offs they are willing to make between components.

o Service users: to identify the trade-offs that client/carers are willing to make in terms of services and access.

## Discussion

Currently there are no models of successful collaborative development of rural allied health service delivery policy. There is no *comprehensive *research evidence (service, staff and client) to support the selection and implementation of any model of allied health service delivery for people with disabilities in rural and remote areas. This project will generate both evidence and the means for ongoing data collection, providing international leadership in this area.

By explicitly considering the work preferences of clinicians with regard to disability service delivery, the outcomes of this project will influence allied health workforce recruitment, retention and deployment practices in rural areas nationally and internationally. This may contribute to alleviating the chronic rural workforce shortages experienced and, in so doing, promote the health of all rural and remote Australians. Equally, by explicitly considering the service preferences of clients and carers, this project will inform the development, focus, delivery and evaluation of disability services in rural and remote areas nationally and internationally.

Finally, this project will promote long-term change in allied health research and service delivery policy in ADHC and other disability services. The overall intent of this project is to institute a sustainable cycle of gathering and applying research evidence to confirm, adapt, develop, implement and evaluate policy to maximise the potential of the allied health workforce to address clients' needs in rural and remote areas.

## Abbreviations

ADHC: NSW Government Ageing Disability and Home Care; ADHC-WR: ADHC-Western region; DCE: Discrete Choice Experiment; ICF: International Classification of Functioning Disability and Health; Non-ADHC: Disability service other than ADHC services; NSW: New South Wales.

## Competing interests

ADHC staff are involved in all aspects of this project. Two authors are ADHC staff.

## Authors' contributions

CV is the principal investigator, led the study's conception and application for funding, chairs the project management group, is involved in all aspects of the study, and drafted the manuscript. ML was involved in the study's conception and application for funding, chairs the project reference group, and is involved in all aspects of the study. AB was involved in the study's conception and application for funding, and is involved in all aspects of the study. GG was involved in the study's conception and application for funding, designed the DCEs, and is involved in all aspects of the study. AD is the project manager, conducted focus groups and interviews, and is involved in all aspects of the study. KB, seconded from ADHC Central Office is the project officer, conducted focus groups and interviews, and is involved in all aspects of the study. JB was involved in the conception and development of the project, contributed to survey design, and is a member of the project working group. SG, Regional Director ADHC-WR, co-led the study's conception, co-chairs the project management group, provides input on disability services and regional characteristics. All authors read and approved the final manuscript.

## Pre-publication history

The pre-publication history for this paper can be accessed here:

http://www.biomedcentral.com/1472-6963/12/70/prepub
